# Evolution of microstructure and related optical properties of ZnO grown by atomic layer deposition

**DOI:** 10.3762/bjnano.4.78

**Published:** 2013-10-28

**Authors:** Adib Abou Chaaya, Roman Viter, Mikhael Bechelany, Zanda Alute, Donats Erts, Anastasiya Zalesskaya, Kristaps Kovalevskis, Vincent Rouessac, Valentyn Smyntyna, Philippe Miele

**Affiliations:** 1 European institute of membranes (IEM, ENSCM-UM2-CNRS, UMR 5635), University of Montpellier 2, Place Eugène Bataillon, F-34095, Montpellier, France; 2 Faculty of Physics, Odessa National I.I. Mechnikov University, 42, Pastera, 65026, Odessa, Ukraine; 3 Institute of Atomic Physics and Spectroscopy & Institute of Chemical Physics, University of Latvia, 19, Raina Blvd., LV 1586, Riga, Latvia,; 4 Institute of Chemical Physics, University of Latvia, 19, Raina Blvd., LV 1586, Riga, Latvia

**Keywords:** atomic layer deposition, optical properties, photoluminescence, thin films, ZnO

## Abstract

A study of transmittance and photoluminescence spectra on the growth of oxygen-rich ultra-thin ZnO films prepared by atomic layer deposition is reported. The structural transition from an amorphous to a polycrystalline state is observed upon increasing the thickness. The unusual behavior of the energy gap with thickness reflected by optical properties is attributed to the improvement of the crystalline structure resulting from a decreasing concentration of point defects at the growth of grains. The spectra of UV and visible photoluminescence emissions correspond to transitions near the band-edge and defect-related transitions. Additional emissions were observed from band-tail states near the edge. A high oxygen ratio and variable optical properties could be attractive for an application of atomic layer deposition (ALD) deposited ultrathin ZnO films in optical sensors and biosensors.

## Introduction

Zinc oxide (ZnO) is an n-type semiconductor and a transparent conductive oxide (TCO) with excellent optoelectronic properties, a wide band gap (3.36 eV), a high dielectric constant, a high exciton binding energy (60 meV), and a high thermal stability [1]. Hence it is an important material for different applications in devices such as gas sensors [2], biosensors [3], transducers [4], solar cells [5–7], electronic and optoelectronic instruments (i.e., ultraviolet photo-detectors) [8], surface acoustic wave (SAW) gadgets [9], and transparent electrodes [10]. ZnO crystals with a grain size in the range of 1–50 nm have demonstrated optical properties, such as an UV shift of the absorption edge and strong photoluminescence at room temperature caused by quantum confinement [11] and, an improvement of the photovoltaic and sensor performance due to a high surface area [12–13]. ZnO nanostructures are obtained as nanoparticles [14], nanotubes [15], nanowires [5,7], and ultrathin films [16–17]. Ultrathin ZnO films can be synthesized by different deposition techniques such as sol–gel [18], chemical vapor deposition [19], electro-deposition [5–7], RF sputtering, and atomic layer deposition (ALD) [16–17].

It is well known that the optoelectronic properties of zinc oxide thin film [20–21] are strongly dependent on the structure [11,22]. Crystallinity and stochiometry of the film determine the concentration of point defects (zinc and oxygen vacancies, interstitial zinc and oxygen) [20]. The band gap of ZnO nanostructures decreases from 3.29 to 3.23 eV with an increase of the grain size [21]. The electrical conductivity of ZnO is affected by a defect concentration and diminishes at annealing in an oxygen environment (oxygen vacancy healing) [21].

One of the methods applied to analyze the crystalline structure and defect level in zinc oxide is photoluminescence. It has been shown that ZnO exhibits a narrow UV emission band in the 378–381 nm range and a broad emission band in the range of 480–620 nm [23–25]. The UV emission band in ZnO has been related to exciton emission, whereas the vis emission has been related to radiative transitions involving intrinsic point defects (O/Zn vacancies and O/Zn interstitials) [23–25].

ALD is an innovative deposition technique which allows depositing ultrathin metal oxide films with preferred thickness, grain size, chemical composition, texture, surface morphology, and defect concentration [26]. The mentioned structural parameters make a strong impact on optical, electrical and additional properties [16–17].

In this paper results of a study on tuning the optical properties (absorption and photoluminescence) along with impacts on grain size, texture, and strain at varying thickness of ultrathin ZnO films are reported. We also discuss our findings with regard to their potential usefuleness for applications in photovoltaics [5,27], photocatalytics [16], sensors [16] and biosensors [28–30].

## Results and Discussion

### Chemical and structural characterizations

ZnO films with a thickness of 25, 49.8, 124 and 250 nm were obtained at 100, 200, 500 and 1000 deposition cycles, respectively. The average growth rate calculated from all results is 2.5 Å per cycle. Results of ellipsometric measurements are presented in Table 1.

**Table 1:** Thickness of ZnO thin films measured by SEM and ellipseometry. Content of Zn and O estimated from EDX analysis.

ZnO number of cycles	Thickness (nm) measured by ellipsometry	Thickness (nm) measured by SEM	O content (atom %)	Zn content (atom %)	O/Zn ratio

100	25	23	66	33	2
200	49.8	45	63	37	1.7
500	124	120	58	42	1.38
1000	250	241	56	44	1.27

**Figure 1:**
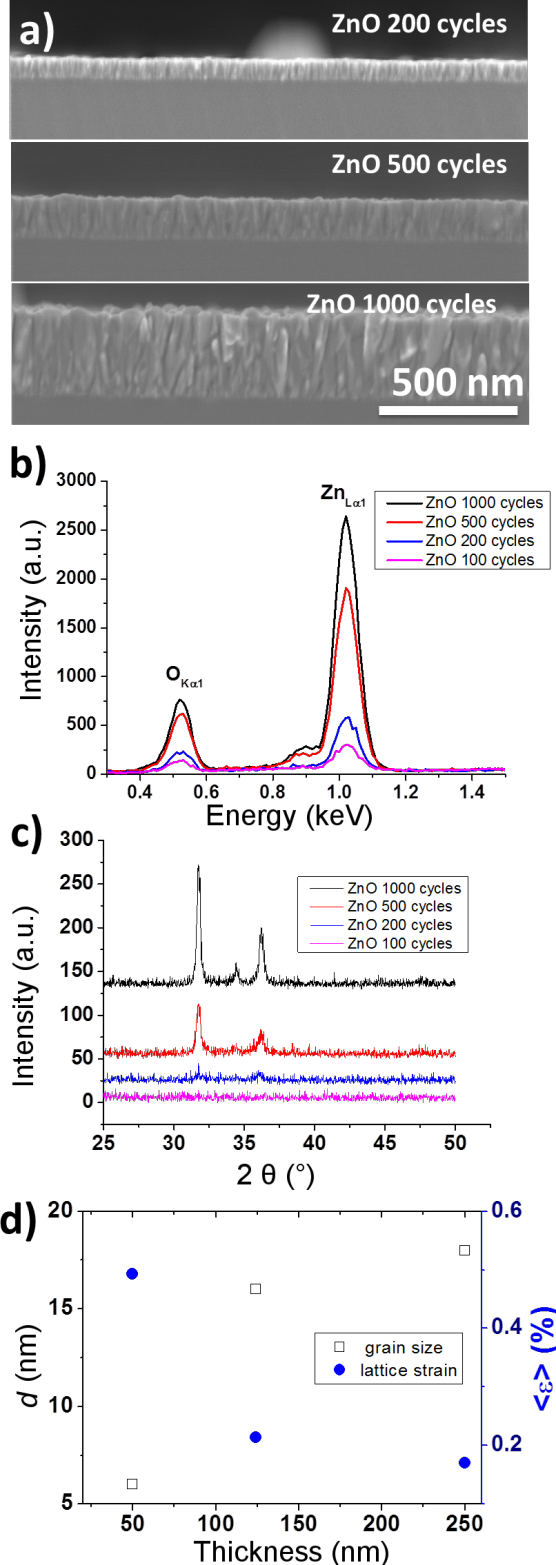
a) SEM images of a cross section of ZnO ALD films deposited on Si substrates by 200, 500, and 1000 cycles; b) EDX; c) GIXRD of ZnO ALD films deposited by 100, 200, 500, and 1000 cycles; d) grain size and lattice strain in ZnO ALD films of different thickness.

SEM images of ZnO thin films grown by ALD on Si substrates at 200, 500, and 1000 cycles are shown in Figure 1a. The images indicate conformal coating of the Si substrate. A rough surface of columnar growth of the films develops with an increase of the film thickness. Cross section images of the same ZnO samples (Figure 1a and Supporting Information File 1, Figure S1) confirm the ellipsometric results.

EDX measurements were carried out to evaluate the chemical composition of ALD ZnO films deposited on Si substrates. The results of these measurements are presented in Figure 1b. An analysis of these results shows that in all the studied ZnO films grown at 100 °C the ratio of O/Zn exceeds 1. This might be caused by the presence of residual OH^−^ and a partially hydroxylated phase ZnO(OH) on the surface of the ZnO grains due to an incomplete removal of excess H_2_O at such a low temperature or a fraction of unreacted hydroxyl groups observed earlier [31–32]. However, as the layers of ZnO are too thin the FTIR results do not confirm the presence of OH groups (Supporting Information File 1, Figure S2). We also did not detect any carbon that remained from the deposition process. Table 1 shows that the O/Zn ratio decreases with increasing thickness.

GIXRD diffraction patterns of thin ZnO films are shown in Figure 1c. The X-ray diffraction from the thinnest samples (25 nm) does not display any peaks. This indicates either an amorphous structure or small (<4 nm) grains [27]. Weak XRD peaks at 2θ = 31.74 and 36.22° corresponding to (100) and (101) reflections of ZnO, respectively, appear in 49.8 nm thick samples and become distinctly pronounced strong reflections in 124 and 250 nm thick samples. An XRD peak of low intensity at 2θ = 34.42° is observed from thick films. Lattice constants calculated from GIXRD spectra of 49.8, 124, and 250 nm thick ZnO films are equal to *a* = 0.325 nm and *c* = 0.52 nm. No significant changes of lattice parameters are observed with increasing film thickness. The maximum values of the texture coefficients (TC) of 49.8, 124, and 250 nm thick ZnO films calculated according to Rivera et al. [33] (1.2, 1.66 and 2.12, respectively) match the preferred growth in the [100] direction. Calculations of the average grain size and lattice strain by the Warren–Averbach techniques with WinFit software show that the growth of the ZnO layers is assisted by a growth of grains and a decrease of lattice strain (Figure 1d).

**Figure 2:**
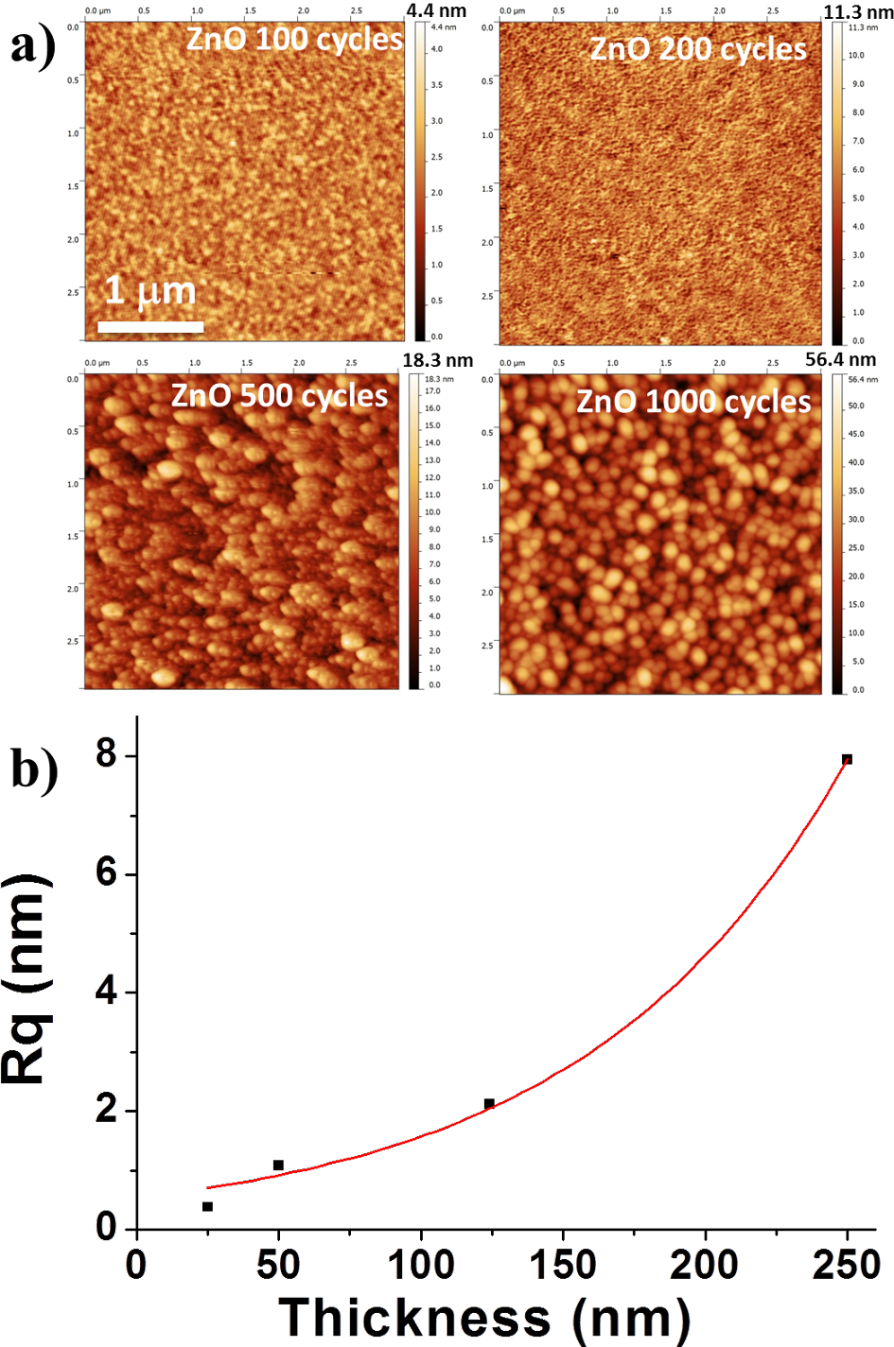
a) AFM images of the size of 3 × 3 μm^2^ of ZnO thin films deposited by 100, 200, 500 and 1000 cycles. b) Roughness as a function of the film thickness.

Surface morphology of the samples was studied by atomic force microscopy (Figure 2a). Samples with a thickness of less than 100 nm have a smooth surface did not feature a significant roughness. Well shaped 100–150 nm elevations are observed on surfaces of thicker samples. The mean-square roughness, calculated from AFM data, has a non-linear (positively accelerated) relationship with the thickness (Figure 2b).

Figure S3 (Supporting Information File 1) shows the TEM cross-sectional analysis of 250 nm thick ZnO. An amorphous ZnO layer is observed at a thickness below 20 nm. No columnar grains are present on the surface of 20 to 50 nm thick films. The grains are mostly randomly oriented thereby indicating that the nano-crystalline grains in the ultrathin (20–50 nm thick) ZnO films are surrounded by amorphous pockets. This finding is consistent with earlier results [34]. The columnar growth is observed in ZnO films thicker than 50 nm. The studied columnar structures have amorphous ZnO surroundings.

Thus, the structure of ZnO ALD films strongly depends on the thickness. The ZnO samples obtained by 100 cycles were amorphous, had a smooth surface, and did not exhibit any XRD peaks. The increase of the film thickness was assisted by the growth of vertically oriented columns with well-defined boundaries, an improvement of the crystalline structure (narrowing of XRD peaks), a crystalline growth and an alleviation of lattice strain, and an enhancement of the surface roughness and the texture coefficient.

### Optical properties

**Figure 3:**
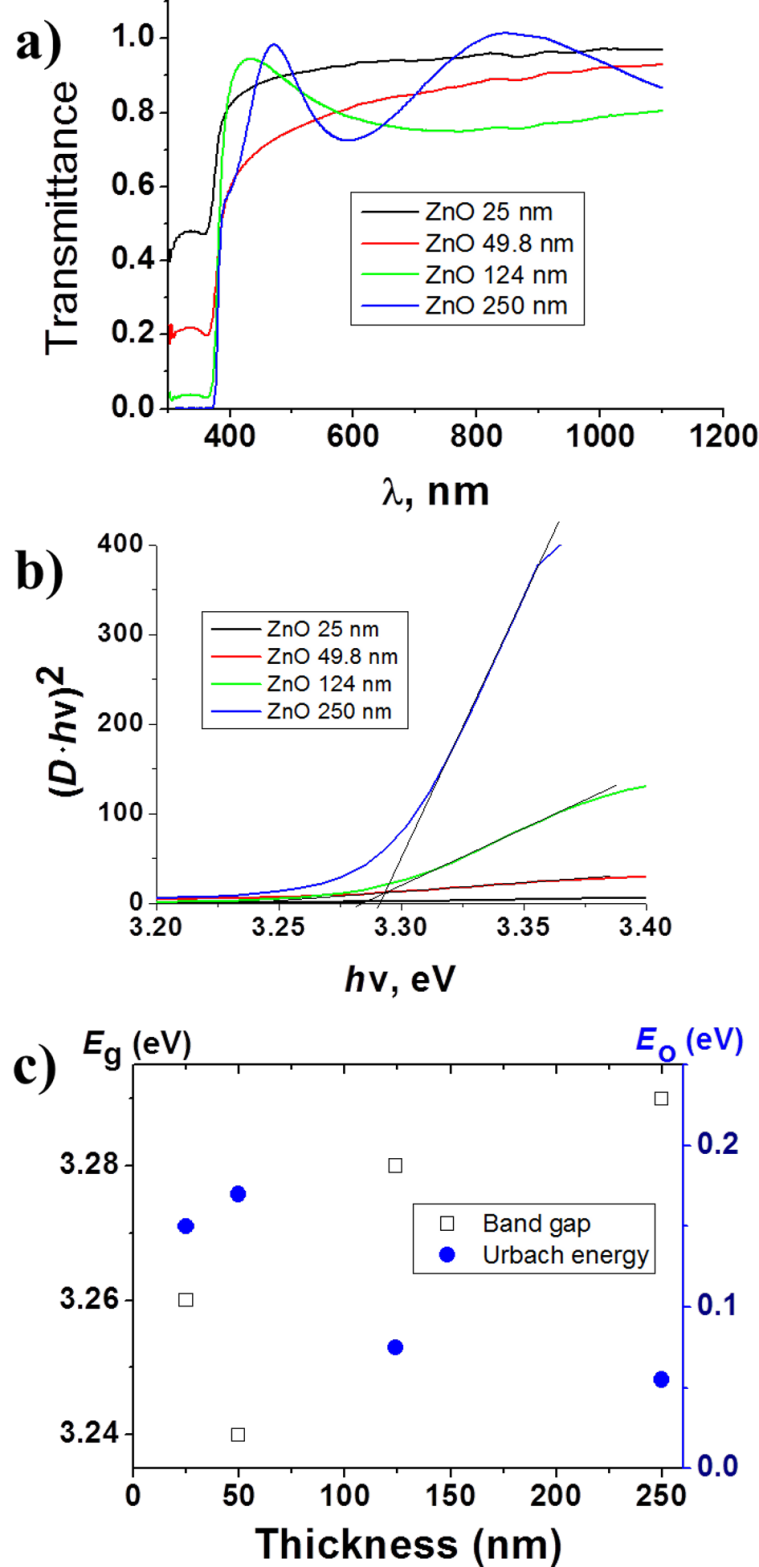
a) Transmittance spectra, b) band gap estimation, and c) band gap and Urbach energies of ZnO ALD films of different thickness.

**Transmittance spectra.** Transmittance spectra of the samples are shown in Figure 3a. ZnO films with a thickness of less than 100nm are transparent in the 500–1100 nm range. Observed transmittance maxima and minima in the spectra of films with a thickness of more than 100 nm match the interference patterns. Since ZnO is an n-type semiconductor of direct optical transitions the optical density *D* is calculated as:

[1]
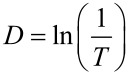


where *T* is the optical transmittance. The optical density *D* is related to the band gap *E*_g_ by proportion [35]:

[2]
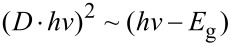


where *h*ν is the photon energy, and *E*_g_ is the band gap. Graphically estimated band gap values of thin ZnO films are shown in Figure 3c. The obtained values are lower than the value typical of a ZnO single crystal (*E*_g_ = 3.37 eV). This difference might be caused by the number of point defects (vacancies and interstitials of Zn and O) [36]. There is a non-typical dependence of the band gap value on the grain size. The small increase of the band gap value with the film thickness may be related to an improvement of the crystalline structure of deposited samples.

The structure of 25 nm thick films is amorphous. The films might contain crystallites with a size of less than 3 nm which are difficult to detect by XRD. In this case, a band gap broadening due to the quantum confinement effect would be expected. However, results obtained from 25 nm films do not show any significant increase of the band gap value. This hints at an absence of small crystallites in the amorphous structure of the films.

Because of the disorder in amorphous and highly doped semiconductors, the absorption or the optical density *D* near the band edge is an exponential function of the photon energy as described by the Urbach law [37]:

[3]
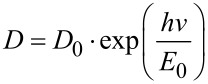


where *E*_0_ is the Urbach energy interpreted as the width of the tail of the states localized close to the conductance band in the forbidden zone. Numerical calculations show a decrease of the Urbach energy while matching the thickness with an advancement of the crystalline structures. This finding correlates well with XRD data.

**Figure 4:**
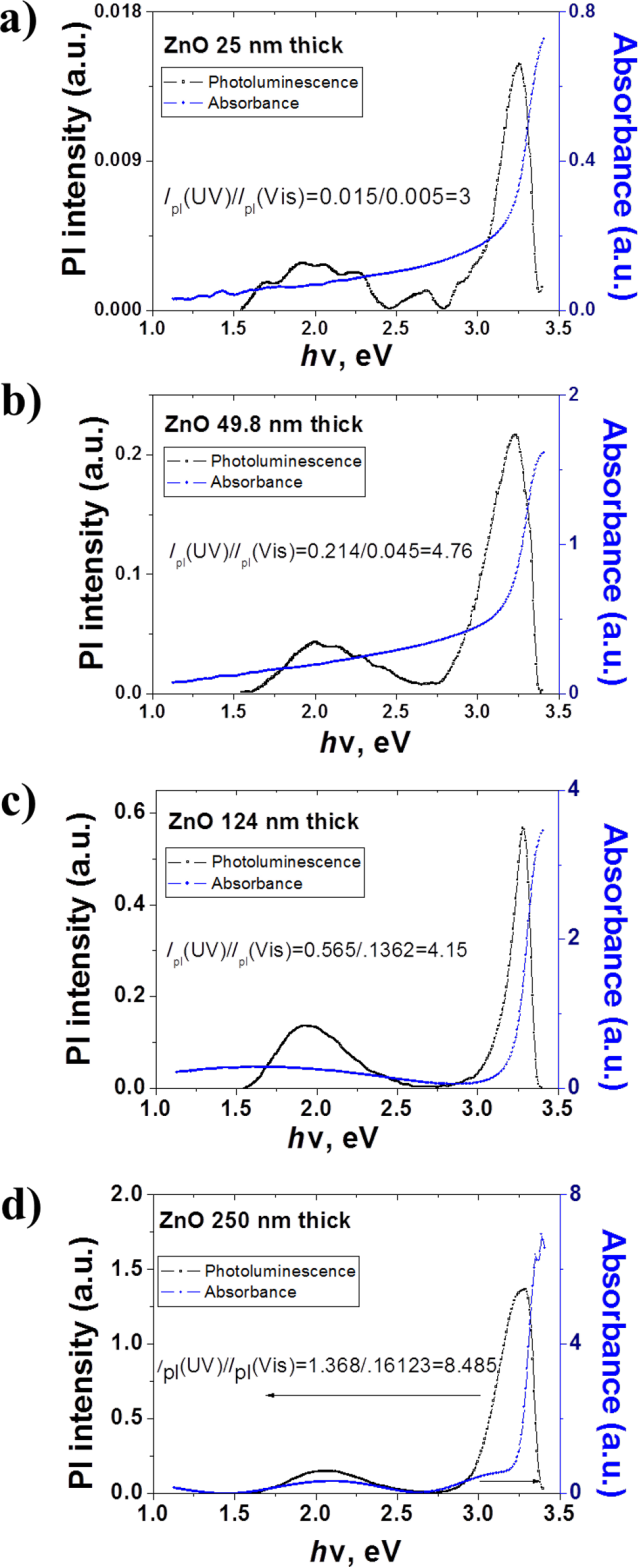
PL and absorption spectra of 25 (a), 49.8 (b), 124 (c), and 250 nm (d) thick ZnO ALD films.

**Photoluminescence and absorption spectra.** The penetration depth of the laser spot in the ALD deposited thin films estimated from transmittance data according to the Beer–Lambert law was about 40 to 44 nm [38]. The latter means that the photoluminescence has an impact from the bulk and the surface of the samples. We suppose that the grain size could play a crucial role in the emitting properties, especially in thicker samples. The 3.08–3.30 eV UV and 1.80–2.28 eV vis emission bands are observed in PL spectra of all ultra-thin ZnO films (Figure 4a–d). The intensity is increasing with the thickness, so it can be controlled by the amount of ZnO material [39].

The shapes of the PL bands fit to a Gaussian peak function by the Origin 7.0 software are presented in Figure S4 of Supporting Information File 1. The main difference between Gaussian and a Lorentz profile is the long tails in the latter case [40] where a lot of the overall intensity is ‘hidden’ in the tails.

Obtained values of the absorption and positions of the PL peaks are shown in Table 2. An analysis of the absorption and the emission shows that the 3.28–3.30 eV PL in the region of the absorption edge is related to band–band transitions [41]. The 3.21–3.24 eV peaks are due to transitions in the band-tail states of ZnO [42]. The observed 3.21–3.24 eV emission belong to electron transitions from tail states of the conduction band to tail states of the valence band [43]. The 3.08–3.14 eV UV peaks correspond to defect states formed by neutral Zn vacancies (V(Zn)0) [44].

**Table 2:** Positions of absorption and PL peaks of ZnO thin films of different thickness.

Thickness (nm)	Peak positions			

	*V*(O^++^) (eV)	(O_i_) (eV)	*V*(Zn^0^) (eV)	Band tail states (eV)

25	2.01103	—	3.08374	3.21019
49.8	1.96819	2.24953	3.09573	3.22453
124	1.88601	2.12068	3.14621	3.24162
250	2.01575	2.24768	3.12942	3.2352

The emission in the visible is caused by point defects [45]. The 2.21–2.25 eV peaks are attributed to oxygen interstitials (O_i_) [44,46–47]. According to Wang et al. [18], emission bands at 1.9–2.0 eV of ZnO ALD ultrathin films are due to doubly ionized oxygen vacancies (*V*(O)^++^) [48]. The PL spectra correlate with the absorption spectra. Absorption spectra have tails and peaks in the 3.2–1.78 eV range matching the optical transitions defect state-valence band and the defect state-conduction band (Figure 4a–d) [49–52]. The binding energy of free exciton in ZnO is 0.06 eV. The exciton emission energy *E*_x_ and the energy gap *E*_g_ are correlated [53]:

[4]
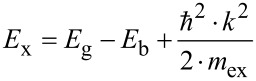


where, *E*_b_ , *ћ*, *k*, *m*_ex_ are the exciton binding energy, Planck’s constant, wave vector, and effective mass of the exciton, respectively. Since ZnO is a direct band gap semiconductor, the wave vector *k* = 0 and the value of the energy gap of the samples, according to Equation 4, is around 3.35–3.36 eV. The difference between the estimations and the experimental data is due to structural defects.

**Correlation between optical and structural properties.** The optical properties of ultrathin ZnO films are tailored by structural parameters (grain size, stoichiometry, etc.). A strong relation between the crystalline structures and photoluminescence of ZnO is described by the intensity ratio of the UV–vis PL bands [20].

In the present study the calculated UV–vis ratio of the PL band intensities of ZnO increase with the growth of film thickness. This may be associated with:

1. improvement of the stoichiometry and the structure of ZnO crystallites
2. grain growth and decreasing number of point effects
3. decreasing bend of the surface band as a result of an increasing grain size.

The structural characterization (GIXRD) shows an improvement of the crystalline structure with the thickness. The EDX results show a monotonous decrease of the O/Zn ratio with a growth of the film while remaining greater than unity, which points to an oxygen-rich stoichiometry of the studied samples. In oxygen-rich films Zn vacancies, oxygen interstitials and oxygen interstates can be formed [46]. The excess oxygen may also localize on grain boundaries to form a negative surface charge [54] and depletion layer. The electric field of the surface charge in the depletion layer would stimulate dissociation of excitons in ZnO [18,48].

The absorption spectra of ultrathin ZnO ALD films show that the defect states are present in the gap. The defect states at 3.08–3.14 eV formed by neutral Zn vacancies and oxygen interstitials at 2.21–2.25 eV identified in PL spectra of oxygen-rich samples correlate well with the EDX results. Doubly ionized oxygen vacancies show up in the PL spectra of all samples at 1.9–2.0 eV pointing to the active role of surface effects in the emission spectra.

**Figure 5:**
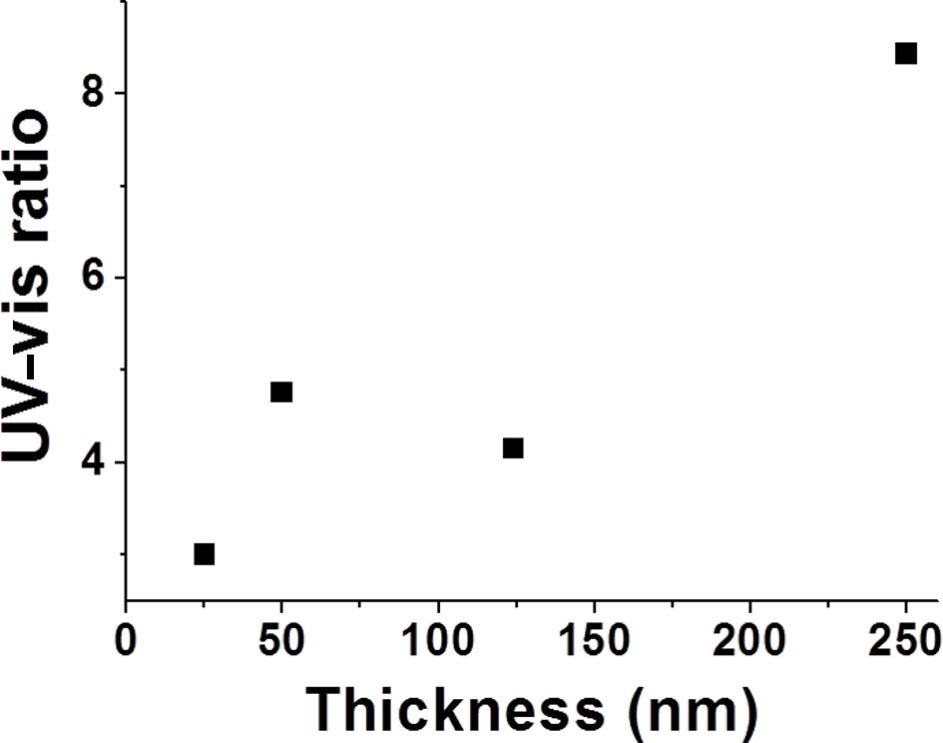
UV–vis intensity ratio of ZnO ALD films of different thickness.

An increasing thickness during the film growth stimulates an improvement of the crystalline structure related to a widening of the energy gap, an increasing intensity of the UV emission, and a decrease of the Urbach energy. An increase of the grain size induced a decrease of the active surface area and affects the concentration of oxygen adsorbed on the grain surface. Therefore, a growing UV–vis intensity ratio in ZnO PL being observed (Figure 5) a decrease of the bending of surface band and the depletion layer is expected if the UV–vis intensity ratio grows in ZnO PL.

The photo-generated electrons and holes are known to be separated by a strong electric field in the depletion region formed by the surface charge [55–56]. The negative charge at the surface and the band bending upward are primarily caused by the adsorbed species (oxygen, hydroxyl groups, etc.) [55–56].

Under steady-state conditions the equilibrium is achieved between the flow of holes to the surface and the flow of electrons to the “bulk” region [56]. The holes reaching the surface reduce the surface charge and, therefore, the bending of the surface band. As a result, the near-surface electric field and the width of the depletion region decrease with increasing intensity of the excitonic PL.

Liao et al. [48] and Wang et al. [18] report that the decrease of the depletion layer in ZnO nanostructures are capable to stimulate transitions between neutral, single-charged, and doubly ionized oxygen vacancies assisted by a UV shift of the visible emission. The neutral oxygen vacancies are located in the bulk, whereas the doubly ionized vacancies are located in the depleted region.

However, in the present study neither the UV shift of the visible emission, nor emission peaks corresponding to neutral oxygen vacancies have been observed. The UV–vis ratio facilitates the discrimination between completely depleted (UV–vis ratio significantly smaller than unity) and partially depleted ZnO grains (UV–vis ratio greater than unity) [18,48]. Since the UV–vis ratio exceeds unity in all measured cases the samples should contain partially depleted grains. Thus, the oxygen vacancies may be formed as point defects mostly in the surface region in an oxygen rich environment, and the concentration in the bulk of the grains may be negligible. Therefore, the change of the width of the depleted layer should not affect emission from oxygen vacancies.

## Conclusion

As the thickness of oxygen-rich ultra-thin ZnO films is grown by atomic layer deposition from 25 nm to 250 nm a structural transition from the amorphous to the polycrystalline state occurs. The increase of the size of the crystalline grains at consecutive deposition cycles is accompanied by a decrease of lattice strain, a rise of the Zn/O ratio, and an uncharacteristic change of the energy gap. This uncharacteristic change of the energy gap is explained by a result of the decreasing concentration of point defects, i.e., an improved crystalline structure. Compared with the bulk ZnO crystals the values of the energy gap of the films are lower due to structural defects. The observed UV and visible photoluminescence emissions in the films correspond to band-edge and defect-related transitions, respectively. Additional UV emissions are observed from band-tail states. The defects related to observed PL bands are identified as neutral Zn vacancies, interstitial oxygen, and doubly ionized oxygen vacancies. The optical properties correlate with the crystalline structure, the point defect concentration, the grain size, and the depleted layer.

An increased intensity of the UV emissions reflects an improvement of the crystalline structure with a growing size of the grains. A narrowing of the depleted layer which does not affect the visible emissions is attributed to a low bulk concentration of oxygen vacancies mainly located on grain boundaries. The oxygen excess is attributed to the formation of Zn vacancies, oxygen interstitials and adsorbed molecular oxygen on the surface of grains. The ultra-thin ZnO ALD films are attractive for optical sensor/biosensor applications due to their high oxygen to zinc ratio and variable optical properties. In addition, the presence of hydroxyl terminals leads to the hydrophilicity of the films and improves the immobilization of selected kinds of bio-molecules, thus increasing the suitability for biosensor applications.

## Experimental

### Synthesis of ZnO thin films by ALD

Diethyl zinc (DEZ) (Zn(CH_2_CH_3_)_2_, 95% purity, CAS: 557-20-0) purchased from Sterm Chemical, a p-type silicon(100) wafer obtained from the Korean MEMC company, ITO substrates from Sigma Aldrich, and glass substrates from RS (France) were used to prepare the samples for this study. In order to remove organic contaminants the substrates were pre-cleaned in acetone, ethanol and de-ionized water for 5 min. A tailored ALD reactor [57] was used for the synthesis of ZnO. The ALD was performed by sequential exposures of DEZ and H_2_O separated by nitrogen purge at a flow rate of 100 standard cubic centimeters per minute (sccm). The regime for the deposition of ZnO consisted of 0.1 s pulses of DEZ, 20 s of exposure to DEZ, 40 s of nitrogen purge followed by 2 s pulse of H_2_O, 30 s exposure to H_2_O, and a final 60 s nitrogen purge. ZnO films with 100, 200, 500, and 1000 ALD cycles were deposited on Si and glass substrates to study the influence of the thickness. The temperature was fixed at 100 °C.

### Characterization

Structural properties of the ZnO films were characterized by scanning electron microscopy (SEM), ellipsometry, energy-dispersive X-ray spectroscopy (EDX), and grazing incidence X-ray diffraction (GIXRD). An Asylum Research MFP-3D atomic force microscope equipped with a commercial silicon tip was operated in the tapping mode to study the surface morphology on images of the size of 3 μm × 3 μm. SEM and EDX characterization of the samples were performed by using a Hitachi S-4800 microscope and EDX on Hitachi S-4500 coupled with a Thermofisher EDX detector, respectively. Thickness of the ZnO films were measured by a Semilab GES5E spectroscopic ellipsometer (of extended visible range: 1.23–5 eV) under conditions of a fixed incident angle of 75° close to the Brewster’s angle of silicon substrate, and variable wavelength between 300 nm and 1 µm. Winelli II software was used to fit the experimental tan(ψ) and cos(δ) data in the full wavelength range by using Cauchy dispersion law and a single-layer ZnO adjusted model to obtain the film thickness. A Bruker D5000 instrument was used for structural GIXRD characterizations.

A Shimadzu UV-1700 spectrophotometer was used to study the optical properties of ZnO thin films by 1 nm steps over the 300–1100 nm range, and photoluminescence in the 370–800 nm range. A solid state LCS-DTL-374QT Nd:YAG 355 nm laser source (Russia) at the intensity of 19 mW/cm^2^ was used to excite the luminescence. Emission spectra were registered by the experimental setup described by Viter et al. [58].

## Supporting Information

File 1 Additional figures.
