# Supplementary material for: Evolution of microstructure and related optical properties of ZnO grown by atomic layer deposition
Source: Beilstein J Nanotechnol. 2013 Oct 28;4:690–8. doi: 10.3762/bjnano.4.78 (PMC3817613; doi:10.3762/bjnano.4.78)
Supplement: File 1 — Additional figures. [file Beilstein_J_Nanotechnol-04-690-s001.pdf]

Supporting Information

for

Evolution of microstructure and related optical properties of ZnO grown by atomic layer deposition

Adib Abou Chaaya[‡1], Roman Viter[‡2,3]*, Mikhael Bechelany[§1]*, Zanda Alute[2], Donats Erts[4], Anastasiya Zalesskaya[2], Kristaps Kovalevskis[3], Vincent Rouessac,[1] Valentyn Smyntyna[2] and Philippe Miele[1]

Address: [1]European institute of membranes (IEM, ENSCM-UM2-CNRS, UMR 5635), University of Montpellier 2, Place Eugène Bataillon, F-34095, Montpellier, France, [2]Faculty of Physics, Odessa National I.I. Mechnikov University, 42, Pastera, 65026, Odessa, Ukraine, [3]Institute of Atomic Physics and Spectroscopy & Institute of Chemical Physics, University of Latvia, 19, Raina Blvd., LV 1586, Riga, Latvia, and [4]Institute of Chemical Physics, University of Latvia, 19, Raina Blvd., LV 1586, Riga, Latvia

Email: Mikhael Bechelany* - mikhael.bechelany@univ-montp2.fr, Roman Viter* - viter_r@mail.ru

* Corresponding author

[§]Phone. +33 4 67 14 91 67, Fax. +33 4 67 14 91 19

[‡]These authors contributed equally to the paper

Additional figures

SEM of ZnO ALD films

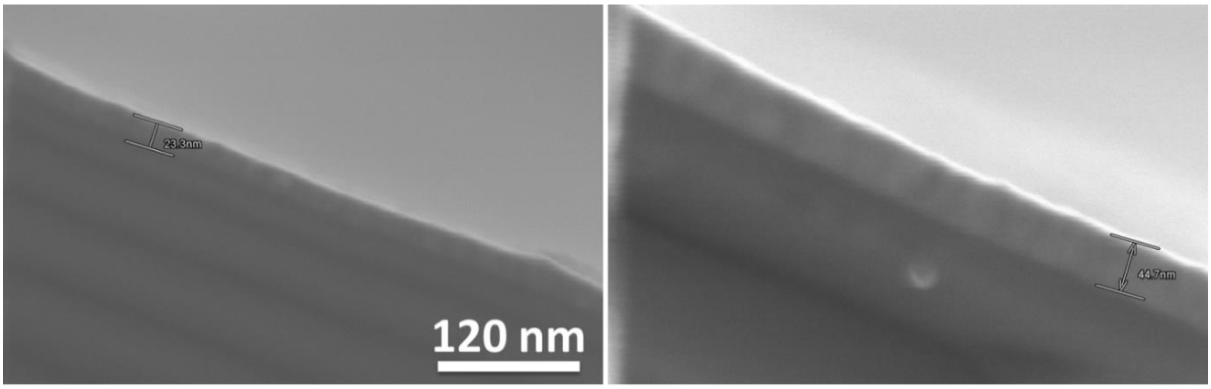

Figure S1: SEM images of the cross section of ZnO ALD films deposited on Si substrates by 100 and 200 cycles.

FTIR spectra of ZnO ALD films of different thickness

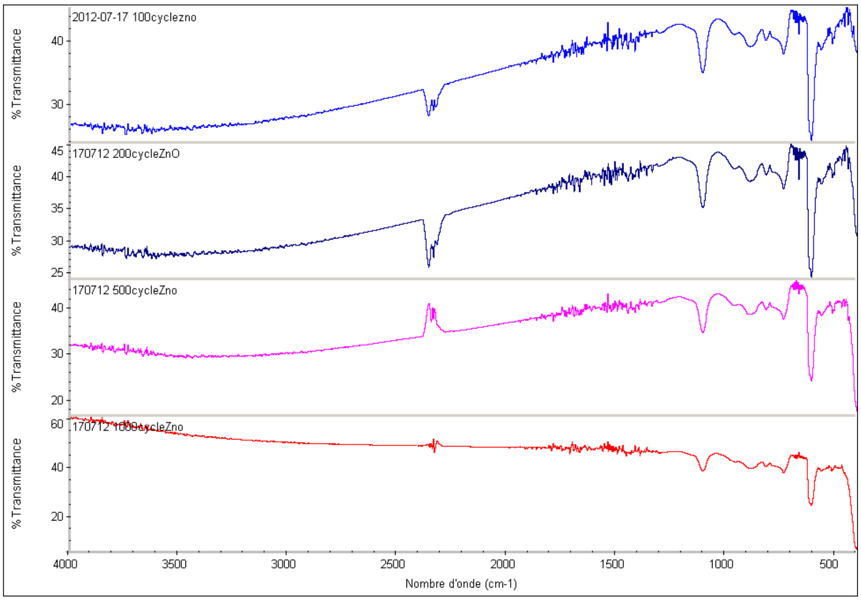

Figure S2: FTIR spectra of ZnO thin films deposited within 100, 200, 500, and 1000 cycles.

TEM measurement of 250 nm ZnO ALD films

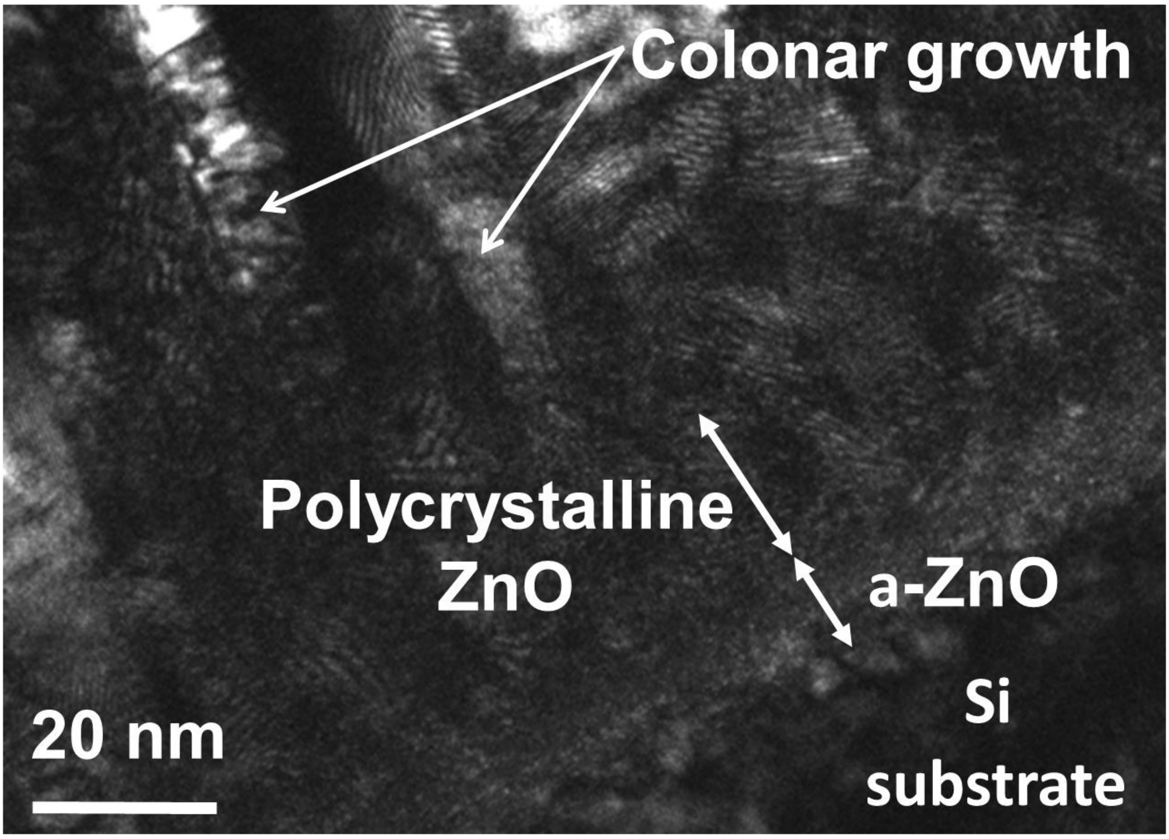

Figure S3: Cross section TEM image of a 1000 cycles ZnO ALD film deposited on Si substrate.

PL measurement of ZnO ALD films of different thicknesses

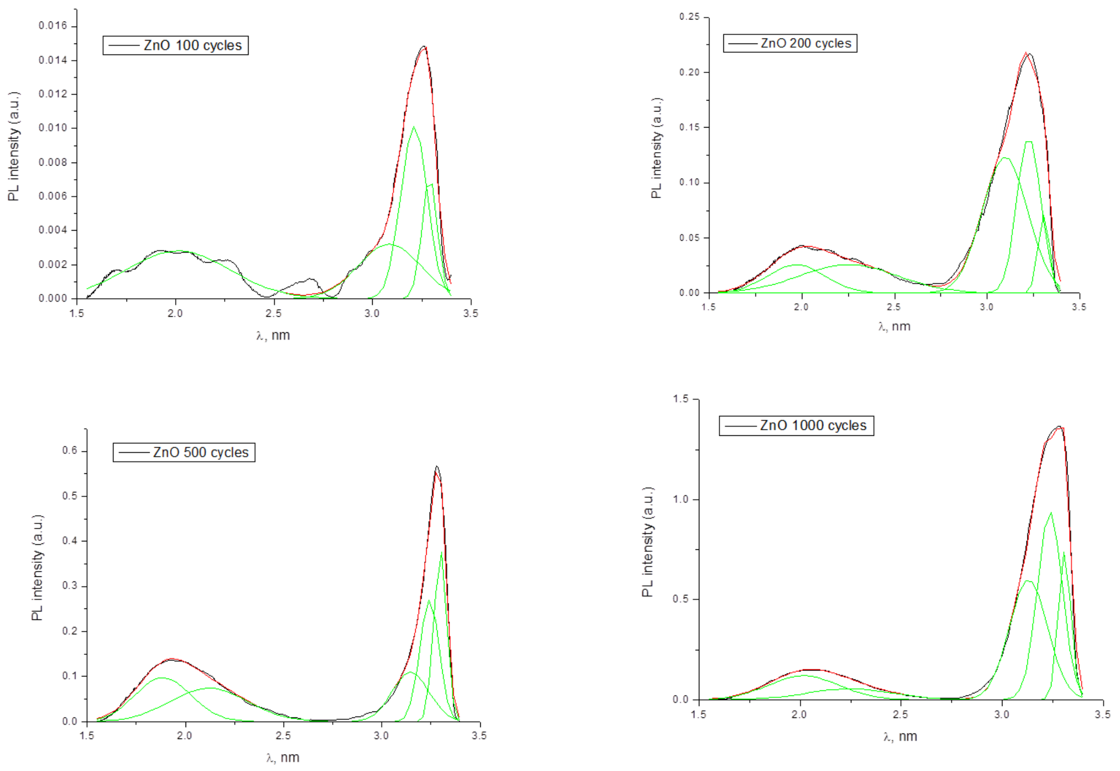

Figure S4: Gaussian fit of PL spectra of 25, 49.8, 124, and 250 nm thick ZnO ALD films.
